# Comparison of two different methods of image analysis for the assessment of microglial activation in patients with multiple sclerosis using (R)-[N-methyl-carbon-11]PK11195

**DOI:** 10.1371/journal.pone.0201289

**Published:** 2018-08-09

**Authors:** Yeona Kang, David Schlyer, Ulrike W. Kaunzner, Amy Kuceyeski, Paresh J. Kothari, Susan A. Gauthier

**Affiliations:** 1 Department of Radiology/Nuclear Medicine, Weill Cornell Medicine, New York, New York City, NY, United States of America; 2 Brookhaven National Laboratory, Upton, NY, United States of America; 3 Judith Jaffe Multiple Sclerosis Center, Weill Cornell Medicine, New York City, NY, United States of America; Macquarie University, AUSTRALIA

## Abstract

Chronic active multiple sclerosis (MS) lesions have a rim of activated microglia/macrophages (m/M) leading to ongoing tissue damage, and thus represent a potential treatment target. Activation of this innate immune response in MS has been visualized and quantified using PET imaging with [^11^C]-(R)-PK11195 (PK). Accurate identification of m/M activation in chronic MS lesions requires the sensitivity to detect lower levels of activity within a small tissue volume. We assessed the ability of kinetic modeling of PK PET data to detect m/M activity in different central nervous system (CNS) tissue regions of varying sizes and in chronic MS lesions. Ten patients with MS underwent a single brain MRI and two PK PET scans 2 hours apart. Volume of interest (VOI) masks were generated for the white matter (WM), cortical gray matter (CGM), and thalamus (TH). The distribution volume (V_T_) was calculated with the Logan graphical method (LGM-V_T_) utilizing an image-derived input function (IDIF). The binding potential (BP_ND_) was calculated with the reference Logan graphical method (RLGM) utilizing a supervised clustering algorithm (SuperPK) to determine the non-specific binding region. Masks of varying volume were created in the CNS to assess the impact of region size on the various metrics among high and low uptake regions. Chronic MS lesions were also evaluated and individual lesion masks were generated. The highest PK uptake occurred the TH and lowest within the WM, as demonstrated by the mean time activity curves. In the TH, both reference and IDIF based methods resulted in estimates that did not significantly depend on VOI size. However, in the WM, the test-retest reliability of BP_ND_ was significantly lower in the smallest VOI, compared to the estimates of LGM-V_T_. These observations were consistent for all chronic MS lesions examined. In this study, we demonstrate that BP_ND_ and LGM-V_T_ are both reliable for quantifying m/M activation in regions of high uptake, however with blood input function LGM-V_T_ is preferred to assess longitudinal m/M activation in regions of relatively low uptake, such as chronic MS lesions.

## Introduction

Multiple Sclerosis (MS) is a chronic inflammatory demyelinating disease of the Central Nervous System (CNS), eventually leading to neurodegeneration. A subset of chronic MS lesions, identified as chronic active or slowly expanding lesions have been described as having a hypocellular lesion center and a rim of activated pro-inflammatory microglia/macrophages (m/M).[1–4] These lesions demonstrate evidence of active demyelination and axonal destruction at their rim and are felt to contribute to long-term, ongoing tissue damage in MS.[1,3,5–8] Chronic active MS lesions have been found to be more prominent in progressive disease and their continued expansion may play an essential role in the pathogenesis of progressive MS.[2,9] Differentiating chronic MS lesion sub-types, especially chronic active lesions and recognizing ongoing biological activity, such as a high degree of inflammation, would provide a unique biomarker for disease activity and ultimately disability progression.

Activated m/M are characterized by marked upregulation of the 18-kDa translocator protein (TSPO) on the cell’s outer mitochondrial membrane.[10–12] Positron emission tomography (PET) can be used to measure the degree of neuroinflammation in MS patients using the radiotracer [^11^C]PK11195 (PK) which targets the TSPO protein.[13,14] Studies have demonstrated a high binding of PK in acute lesions,[15,16] which is consistent with histopathological studies demonstrating diffuse immune cell infiltration during the early stages of lesion development as compared to the lower levels found in chronic MS lesions.[17] The relatively low level of m/M activation in chronic MS lesions presents a challenge for longitudinal detection of change and requires a sensitive analytic method.

The gold standard for analysis of dynamic PK studies is a two-tissue model using a metabolite corrected plasma input function.[12,18] Routine arterial sampling in the clinical setting is not usually feasible; an image-derived input function (IDIF) has been used as a stand-in for arterial blood sampling. Using an IDIF, the Logan graphical method (LGM) has been shown to give reliable results.[19] As an alternative, reference tissue models have been developed that do not require estimation of the radiotracer concentration in blood.[12,13] There have been several reports using a reference tissue model that selects reference voxels based on a supervised cluster analysis algorithm (SuperPK) with four kinetic classes to extract the reference tissue input function.[13,20–23] Although individual clinical studies have utilized either IDIF or SuperPK to assess TSPO radiotracer uptake in MS, no studies have compared their accuracy as it relates to the three main challenges inherent to evaluating m/M activity in MS. The first is that the level of m/M activation can be low, which leads to a low signal-to-noise ratio in the PET image and results in a greater uncertainly in the measurement due to statistical fluctuations. The second factor is the size of the lesion or area of interest; small regions have large influence of partial volume effects, resulting in underestimation of the level of activation. Since lesions can change in both size and inflammatory activity over time, these issues need to be addressed for accurate longitudinal comparisons. The third factor is the presence of widespread m/M activation, studies have demonstrated the presence of m/M within normal and gray matter,[24] thus potentially limiting the accuracy of reference-based analytical methods.

Our goal is to determine which PK modeling method would provide the most robust and accurate measure of m/M activation (V_T_ and/or BP_ND_) for chronic MS lesions in longitudinal studies. To do this, we compared the variability of V_T_ and BP_ND_ among CNS regions of varying sizes and levels of PK uptake to assess the effects of statistical uncertainly and partial volume effects related to detecting longitudinal change of m/M activity in chronic MS lesions. We further confirmed our results in a subset of chronic MS lesions.

## Methods

### Human subjects

Ten MS patients underwent a brain MRI and two [^11^C] PK11195 PET (PK PET) scans using a test-retest paradigm with a two-hour interval between scans. The institutional review board (IRB) of Weill Cornell Medical College approved all procedures and all subjects gave written informed consent. Patients were selected from two ongoing longitudinal PK PET studies in relapsing and progressive MS patients. Both studies were approved by an ethical standards committee on human experimentation and written informed consent was obtained from all patients. Baseline patient test-retest PK PET data was utilized for this analysis. Patient characteristics and clinical data were obtained within 1 month of the individual’s brain MRI and PK PET scan (Table 1).

**Table 1:** Subject characteristic.

Subject	Gender	Age	DT	DD	EDSS
1	M	41	PPMS	15	6.5
2	F	25.5	RRMS	4	4
3	F	61	RRMS	15	2
4	F	50	RRMS	3	0
5	M	43.5	RRMS	5.7	1
6	M	25	RRMS	1	1
7	F	57	SPMS	11	5.5
8	F	47.5	RRMS	22	3
9	M	58.9	RRMS	4.1	1
10	F	30	RRMS	8	1

Clinical characteristics of the subjects participating in the study, DT = disease type, DD = disease duration (in years), EDSS = expanded disability status scale, F = female, M = male, RRMS = Relapsing Remitting MS, PPMS = Primary Progressive MS, SPMS = Secondary Progressive MS.

### Radiopharmaceutical

The radiopharmaceutical, (R)-[N-methyl-^11^C]PK11195 (PK), was prepared by modifying previously reported synthetic procedures.[25,26] The radiotracer was prepared by dissolving 2 mg of des-methyl (R)-PK11195 in 350 μL DMSO and vigorously mixing with 3.2 μL 5M sodium hydroxide. Following collection of [^11^C]-methyl iodide into DMSO solution, the mixture was heated at 800°C for 5 min. The product mixture was subjected to HPLC purification and the desired fraction containing (R)-[N-methyl-11C]PK11195 was isolated and reformulated in saline containing 7% ethanol. The average specific activity at the end of bombardment (EOB) was ~1500 GBq/μmol (40.6 ± 24.5 Ci/μmol) and the synthesis took 42 ± 2 min.

### PK Administration

All doses were diluted in saline to produce a final volume of 10 mL with no adjustment in dose for body weight. Doses were infused over 60 seconds with an automated pump in a “slow bolus” paradigm. The net injected doses averaged 419 ± 52 MBq (11.3 ± 1.4 mCi) for the test condition, and 375 ± 78 MBq (10.2 ± 2.1 mCi) for the retest condition. There was no significant difference in injected radioactivity dose or injected mass between test and retest scans (p = 0.18).

#### Image acquisition and processing

All PET images were acquired with the same time-of-flight whole body PET scanner (mCT, Siemens/CTI, Knoxville, TN).[27] All PET images were corrected for photon absorption and scatter using an in-line CT scanner set at 120 kV, a pitch of 1.5, and 30 mA, and for random using the delayed coincidence window subtraction method. The axial FOV is 16.2cm (with the 3 rings of this PET scanner). PET data were reconstructed in a 400×400 matrix with a voxel size of 1.082×1.082×2.025 mm^3^ using a zoom of 2.0 and an iterative-plus-time of flight (+ TOF) list-mode reconstruction algorithm provided by the manufacturer using ordered subset estimation maximization (OSEM) methods with 4 iterations and 21 ordered subsets. Tissue concentrations were estimated by reconstructing the list mode data into 22 frames (4 frames of 15 seconds (s) each, then 4×30s, 3×60s, 2×120s, 8×300s and 1×600s) for a total scan time of 60 minutes.

### Magnetic resonance imaging (MRI)

MR imaging was performed at 3 Tesla using a Siemens Magnetom Skyra scanner (3.0 Tesla TIM TRIO MRI system, Siemens Healthcare; Cary, NC). Obtained sequences included T1w BRAVO/ Magnetization-Prepared Rapid Gradient-Echo (MPRAGE) (0.94x0.94x0.94 mm3) with and without gadolinium enhancement, T2w (0.5x0.5x3 mm3), and T2-FLAIR (1x1x1 mm3) images were obtained. For the MPRAGE acquisition protocol a 32 channel head coil was used, to provide optimal gray matter/white matter contrast. A spatial resolution of 1.0 mm x 1.0 mm x 1.0 mm was used with a 256 x 256 matrix, 160 slices, and 2170 ms/4.3 ms/1100 ms TR/TE/TI times with an acceleration factor of 2 and a flip angle of 7°.

### Volumes of interests (VOIs)

Automated brain segmentation was performed on the MPRAGE MRI data using FreeSurfer v8.0 (Martinos Center for Biomedical Imaging, Charlestown, Massachusetts). Summed PET images were coregistered to their corresponding MRI scans using rigid registration with mutual information in PMOD® (PMOD Technologies Ltd., Zurich Switzerland).[28] Each subject’s own freesurfer segmentation of the cerebral VOIs, including the thalamus (TH), cortical gray matter (CGM) and white matter (WM), was also coregistered to the PET images. To investigate the influence of size on the parameter estimates, different sized VOI masks were created for the WM and TH by first defining the boundaries of the region using the FreeSurfer defined edges. The boundaries of the VOI were systematically moved to the center of the region in steps of one voxel until the VOI was 1 cm^3^. Mean of VOIs with ten subjects were shown in Table 2.

**Table 2:** Volume of interests—Volume information.

**Volumes** **[CCM]**	Region	TH	WM	GM			
	AVG	14.02	446.61	467.58			
	SD	2.12	78.30	89.09			
	CoV(%)	15.15	17.53	19.05			
	Region	TH-1	TH-2	TH-3	TH-4	TH-5	
	AVG	11.36	7.58	5.34	3.28	1.49	
	SD	1.03	0.57	0.40	0.49	0.33	
	CoV(%)	9.06	7.49	7.57	14.90	22.33	
	Region	WM-1	WM-2	WM-3	WM-4	WM-5	WM-6
	AVG	290.71	142.95	70.25	31.78	7.36	3.09
	SD	59.75	27.19	12.14	5.09	1.73	1.51
	CoV(%)	20.55	19.02	17.28	16.02	23.55	48.97

It is shown mean(AVG) and standard deviation(SD) of volume of interests with 10 subjects. Abbreviation: Cov(%): coefficient of variation; TH: thalamus; WM: white matter; GM: gray matter.

### Lesion region of interests

White matter (WM) segmentation with FreeSurfer [29,30] and a semi-automated lesion mapping method was used on the acquired T1 and T2 data. First, the WM and grey matter (GM) masks were segmented from the T1 images and were checked and edited for misclassification due to WM T1-hypointensities associated with lesions.[31] The WM hyperintensity lesion masks were created from the T2 FLAIR images by categorizing the tissue type based on the image intensities. They were then masked with the segmented FreeSurfer WM volume to include only WM abnormalities. All the images were aligned onto FreeSurfer volume with the boundary-based registration. The WM lesion masks were overlaid on T2 and T2 FLAIR images and a trained neurologist (SG) gave a final approval on edits.

### Image-derived input function (IDIF)

Circular ROIs of 4 mm in diameter were drawn around the carotid arteries at the C4 level. Two Circular ROIs of 8 mm in diameter were placed about 2 cm from the arteries in the normal appearing white matter of the bilateral anteromedial temporal lobes to assess any background spill in.[32] The ROIs were checked on every PET frame using landmarks in the images to ensure that any head movement did not result in misplacement. PK is greater than 98% plasma protein bound and thus we assume that whole blood is an accurate approximation for plasma.[33] Previous work indicates that the ratio of whole blood [^11^C]PK11195 to plasma does not change over the 60 minutes of the PET scan, therefore a population-based average of the metabolite profile was used for this analysis.[33]

### Measurement of distribution volumes (V_T_)

A time activity curve was extracted for each CNS tissue region. The Logan graphical method (LGM)[34,35] was used to calculate the total distribution volume (LGM-V_T_) in each VOI for both the test-retest studies using t* = 30 min and a constant weighting factor. The concentration of the radiotracer in the whole blood was analyzed as a linear interpolation of the concentration before the peak and a three-component exponential fit of concentrations after the peak. All kinetic analyses were performed using PMOD 3.5.[27]

### Measurements of non-displaceable binding potential (BP_ND_)

The reference Logan graphical model (RLGM)[36] was used to calculate BP_ND_. A computational supervised clustering methodology (SuperPK) was used for extracting a reference curve on PK-PET.[19, 20]

### Test-retest reliability

To evaluate the test-retest reliability of [^11^C]-PK11195 binding parameters, the percent of the test-retest variability (TRV) of both V_T_ and BP_ND_ were calculated for each subject, i.e. $TRV=100*\left\{\frac{T-RT}{\frac{\left(T+RT\right)}{2}}\right\},$ where T and RT refer to values from the test and retest scans, respectively. The mean TRV will indicate any systematic trend of [^11^C]-PK11195 binding between the test and retest scans with a value of near zero indicating no systematic trend. The standard deviation (SD) of TRV will reflect uncertainty in the percent change between the two scans.

Descriptive statistics of precision and bias included means, SD, repeatability coefficients (RC) and the mean of the absolute value of TRV (aTRV). The RC values were calculated because they are useful indices that quantify absolute reliability measurement error in the same units as the measurement tool.[37–39] The RC is an interval beyond which the absolute differences between two measurements have a higher than 0.95 probability of representing a true change. Both random and systematic errors are taken into account by the RC score.[40] The %RC is calculated using the following equation[37]:

$$\%RC=100*2.77*\sqrt{\frac{1}{P}\sum_{j=1}^{P}\frac{\frac{{\left({VT}_{test}-{VT}_{retest}\right)}^{2}}{2}}{{\left(\frac{{VT}_{test}+{VT}_{retest}}{2}\right)}^{2}}}$$

where P is the number of test-retest pairs. The descriptive statistics and computations for test-retest analysis were performed using SPSS (IBM Corp. Version 22.0. Armonk, NY: IBM Corp). In all analyses, the statistical significance (alpha level) was set at p<0.05.

## Results

### Time activity curves with VOI size

**Fig 1:**
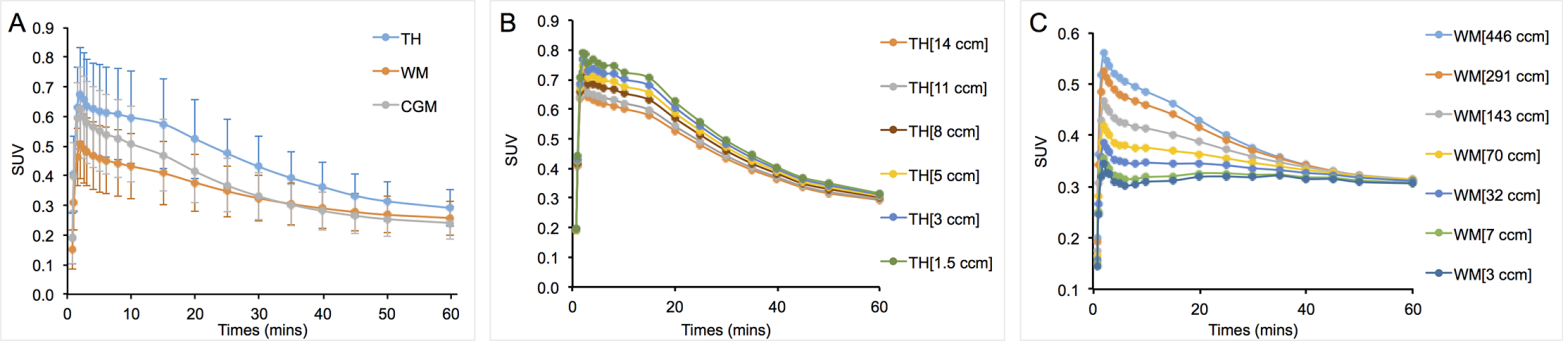
Time activity curves in various ROIs. (A) Mean tissue time-activity curves expressed as standardized uptake values (SUV) for 20 scans in 10 subjects. (B) Mean tissue time-activity curves of various volumes within the thalamus, expressed as standardized uptake values (SUV) for 20 scans in 10 subjects. (C) Mean tissue time-activity curves of various volumes of white matter expressed as standardized uptake values (SUV, 20 scans in 10 subjects).

The mean TACs for three regions of interest (TH, WM and CGM) in the 20 test-retest scans are shown in Fig 1A. The mean TACs with the various sizes of VOIs in the TH and WM are shown in Fig 1B and 1C. The TH, which had the highest PK uptake, showed an increased observed tracer uptake as the size of the VOI decreased (Fig 1B). However, WM, which was the lowest uptake region, showed a decrease in the uptake as the volume decreased (Fig 1C).

### Evaluation of the image-derived input function (IDIF)

**Fig 2:**
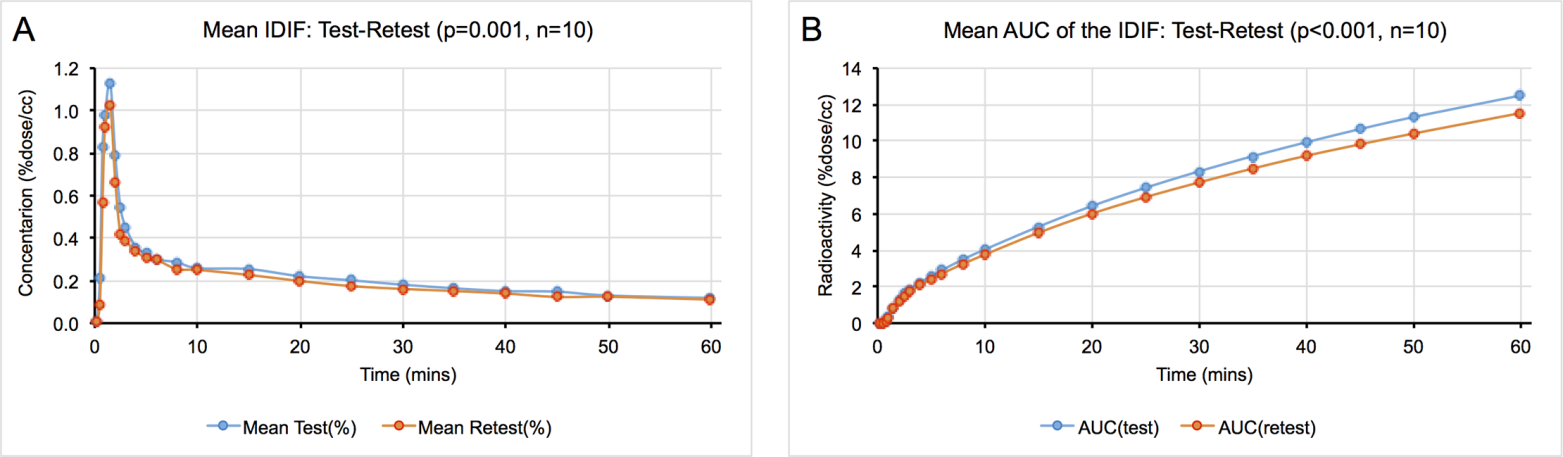
Image-derived input function characteristics. (A) Time activity curves of the image-derived input function with test-retest studies were shown. There was no significant difference (p = 0.001). (B) It was shown area under the curves of IDIF with test-retest studies and was not shown any significant difference.

Image-derived input functions were used to establish the concentration of radioactivity in the blood. The mean TAC of test scans and retest scans showed high repeatability (p = 0.0012, Fig 2A). The value of the area under the curve (AUC) was computed using three different time intervals (20–60 mins, 40–60 mins, and 1–60 mins) and the ratio between test and retest was calculated (Fig 2B). Every subject showed a standard deviation ranging from 6% to a 9% (Table 3).

**Table 3:** The ratio of area under the curves.

AUC Ratio (test/retest)	AVG	SD	CoV(%)
AUC: 20–60 min	1.09	0.10	9.6
AUC: 40–60 min	1.09	0.09	8.4
AUC: 1–60 min	1.07	0.06	5.7

It is shown the ratio of area under the curves with different times. (n = 10) The ratio is calculated by AUC of test curves divided by AUC of retest curves.

### Test-retest variability of LGM-V_T_

**Fig 3:**
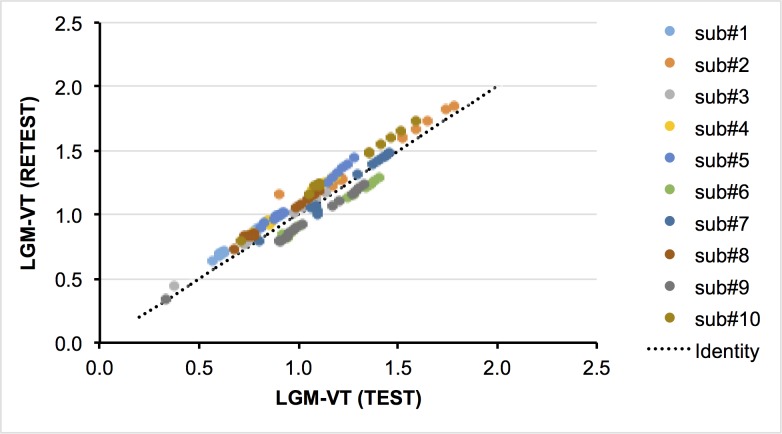
Distribution volume differences with test-retest studies (n = 10). Illustrate correlation of values of LGM_V_T_ between test and retest.

Values of V_T_ in the test scan were lower than those of the retest scan in seven of ten subjects, but the difference between the two scans was not significant using a two tailed T-test (p = 0.28, Fig 3). The mean TRV in all VOIs ranged from -4.48% to -3.46% for the LGM-V_T_ both TH and WM. The mean of the absolute test-retest variability for both TH and WM was not sensitive to the size of the VOI, but did show slight sensitivity to the levels of PK binding. The %RC values ranged from 15 to 21. (See Table 4)

**Table 4:** V_T_ and BP_ND_ values for all VOIs.

VOIs	LGM					RLGM				
	Mean aTRV (%)of VT	SD aTRV(%) of VT	Mean TRV(%) of VT	SD TRV(%) of VT	%RC	Mean aTRV (%)of BPND	SD aTRV(%) of BPND	Mean TRV(%) of VT	SD TRV(%) of BPND	%RC
TH (14 ccm)	7.70	3.35	-3.96	7.73	16.32	2.14	1.40	0.46	2.68	4.88
WM (447 ccm)	9.08	3.31	-4.48	8.96	18.81	6.85	5.92	-4.85	7.95	17.10
GM (468 ccm)	8.37	3.44	-4.48	8.21	17.60	8.01	5.36	-0.26	10.28	18.39
WM-1 (290 ccm)	9.21	3.29	-4.39	9.15	19.05	7.56	5.29	-5.80	7.49	17.56
WM-2 (143 ccm)	9.41	3.23	-4.15	9.47	19.38	7.88	5.36	-6.44	7.31	18.17
WM-3 (70 ccm)	9.64	3.11	-4.00	9.75	19.75	7.65	9.25	-6.52	10.23	22.31
WM-4 (32 ccm)	10.18	2.95	-3.67	10.43	20.67	10.36	13.65	-7.79	15.57	31.74
WM-5 (7 ccm)	10.07	2.73	-3.46	10.34	20.37	18.62	21.45	-11.92	26.56	52.93
WM-6 (3 ccm)	10.42	3.34	-3.97	10.69	21.34	60.27	101.21	-54.71	104.93	216.07
TH-1 (11 ccm)	7.72	3.25	-3.98	7.69	16.28	1.74	1.52	0.44	2.39	4.35
TH-2 (8 ccm)	7.64	3.27	-3.97	7.62	16.15	2.17	1.37	0.49	2.70	4.91
TH-3 (5 ccm)	7.58	3.30	-3.99	7.55	16.06	2.75	1.28	0.45	3.24	5.85
TH-4 (3 ccm)	7.44	3.39	-3.99	7.43	15.88	3.21	1.49	0.41	3.79	6.82
TH-5 (1.5 ccm)	7.29	3.62	-4.12	7.31	15.79	3.87	2.67	0.01	5.01	8.96

### Test-retest variability of BP_ND_ with reference curves generated by SuperPK

Among the 10 patients, extraction of reference region curves failed in three subjects who had extremely low time activity curves in both test-retest scans. In a fourth subject, there was essentially no difference between the WM TAC curve and the reference region TAC, and therefore the supervised cluster analysis failed. Therefore, these four subjects were removed from the final analyses.

**Fig 4:**
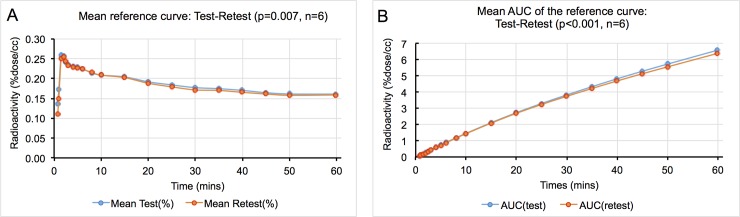
Reference tissue curve characteristics. (A) Mean TAC curves for the reference curves between test-retest studies (n = 6). (B) Mean AUC of the reference curves between test and retest.

The remaining six subjects were evaluated using the generated cluster analysis reference curves. The mean TAC of the reference curves in test scans and retest scans showed excellent repeatability (p = 0.007, see Fig 4A). There was no significant difference in the AUC for test and retest and differences were in general smaller than those observed for the IDIF AUC between test and retest (Fig 4B).

There was excellent repeatability for all sizes of VOIs for the TH (less than 1%) However, in WM, both the mean TRV and aTRV indicated very high values (up to 55%, see Table 4). The %RC was lower compared to LGM-V_T_ values in the TH, conversely the WM %RC values were much higher (up to 200%, Table 4).

### Test-retest variability among chronic MS lesions

**Fig 5:**
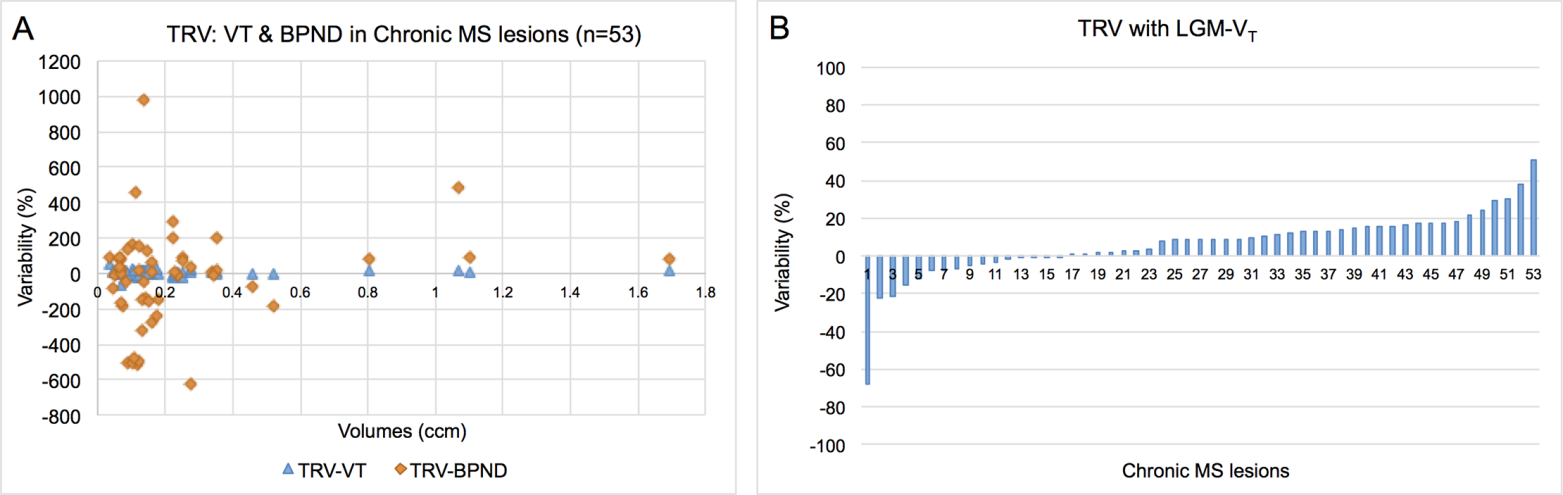
Test-retest variability of chronic lesions in MS patients (n = 53). (A) Graph of the test-retest variability of the V_T_ versus BP_ND_ measures of PK in chronic MS lesions. (B) It shows the percent variability of the LGM-V_T_ measures of PK in 53 chronic MS lesions.

To further test our methods, we identified 53 chronic lesions with volumes ranging from 0.08 to 1.691 cm^3^ (see Fig 5A). In 85% of the lesions the TRV of LGM-V_T_ w was less than 20% (Fig 5B), however the range was much broader for BP_ND_ with LRGM, only 15% of lesions had a TRV value less than 20% (Fig 5C).

## Discussion

Chronic active or slowly expanding MS lesions have been described as having pro-inflammatory microglia localized at the lesion rim and their continued expansion may play an essential role in the pathogenesis of disease progression.[6,12] In those MS patients, where size and activity of both enhancing and chronic lesions may be changing, it is important to determine which method gives the most reliable results under varying conditions. This is a particularly pressing issue for longitudinal comparison studies. The two non-invasive methods we examined are widely used to assess m/M activation in several neurological diseases. Our goal was to determine which PK modeling method would provide the most robust and accurate measure of m/M activation for chronic MS lesions in longitudinal studies.

To accurately determine the level of m/M activation using PET, we need to discern the amount of radiotracer bound in the tissue as determined from the brain VOI, and the amount of radiotracer available in the blood, as determined by either an input function or a reference region. Since the VOIs of the brain regions are the same for both models, it is important to understand how the SUV value of the VOI, containing the lesion, is influenced by both the partial volume effects of spill out and the statistical effects of the number of counts from the region in each of the models. In general, for high activity regions, as the VOIs get smaller, the SUV value increases and the statistical uncertainty is relatively stable. On the contrary, in low activity regions, as the VOIs get smaller, SUV value decreases (Fig 1B and 1C) and the statistical uncertainty increases.

In the Logan graphical IDIF model, a region drawn around the carotid artery is used to determine the level of radioactivity in the blood. One advantage of this method is that the count rate is relatively high soon after injection and there is a very low background. This results in low statistical uncertainty during the time when the radiotracer is actively binding to the tissue. However, one limitation of this method is that the measured level of activity in the blood is inaccurate due to partial volume effects. In almost all PET/CT scanners, the apparent level of radioactivity in the blood will be considerably underestimated given that the size of the carotid is typically 4 to 5mm while the size needed to eliminate the partial volume correction is usually greater than 10mm. Although this has an effect on the absolute value, it does not alter the results obtained for the LGM in comparison to the BP_ND_.

The SuperPK method suffers from the limitation that the generation of useful reference region time-activity curves using the supervised cluster algorithm is not always possible. Supervised cluster analysis requires a set of predefined kinetic classes, which are generated from manually defined ROIs on selected PK PET scans.[41] The relative shape differences between the kinetic classes is important in defining the reference region. Therefore, if there is widespread activation, fewer and fewer voxels will be included in the reference region TAC, increasing the statistical variation. In our cohort, we were unable to extract a reference region curve for four out of ten of the subjects using SuperPK. Although we did not identify a specific reason for this failure, we hypothesize that the clustering algorithm could not separate enough reference region voxels from the other kinetic classes to give a reliable reference region time-activity curve.

The two methods show different sensitivities to the effects of partial volume and statistical uncertainty. As shown in Table 4, repeatability of LGM-V_T_ was high with the various VOIs for both the high activity and low activity regions. In the case of the SuperPK analysis, repeatability was very robust and highly repeatable no matter what size of VOIs is for high activity regions. In fact, the repeatability coefficients (%RCs) were better than LGM-V_T_ (see Table 4). However, the repeatability in the low activity regions (in WM) varied with the size of the VOIs. In small VOIs, the statistical uncertainty of the brain activity curves becomes more important when calculating the values of BP_ND_. When a somewhat noisy reference region curve is combined with the rather noisy TAC from the low activity brain region, the value of BP_ND_ becomes highly volatile.

To further confirm our hypothesis that LGM-V_T_ should be more reliable in assessing MS chronic lesions over time, we compared the variability of the two methods using the test-retest paradigm on actual patients’ MS lesions. Most of the chronic lesions in MS are located in the white matter, which is known to have low PK activation. Fig 5 clearly demonstrates that V_T_ has a substantially higher reliability than BP_ND_ for lesions of all sizes. Therefore, we can safely conclude that using the Logan graphical model with an IDIF will provide a more reliable measure of microglial/macrophage activation in chronic MS lesions.

## Conclusion

Treatments targeting modulation of CNS inflammation would provide a novel therapeutic strategy to reduce tissue injury, neuronal degeneration and clinical disability.[12] Thus, utilizing accurate and robust metrics with which to identify and longitudinally assess chronic active MS lesions will facilitate treatment studies that aim to target chronic MS lesions with higher levels of m/M activity. In this study, we demonstrated that IDIF (V_T_) provides a more robust and accurate measure of m/M across various tissue types as compared to BP_ND_. The results from this study highlight the importance of carefully considering the reliability of different PK PET based kinetic measures in the context of partial volume and statistical effects, particularly when applying these methods to pathological data for longitudinal studies. The analysis applied here should also apply to other radiotracers with a low signal to noise ratio as well as to later generation TSPO radiotracers with a better signal to noise ratio. The SuperPK method may be more robust with these later generation tracers.
